# Amniotic Fluid Embolism After First-Trimester Abortion

**DOI:** 10.7759/cureus.24490

**Published:** 2022-04-26

**Authors:** Subrat Panda, Ananya Das, Nalini Sharma, Rituparna Das, Dr vinayak Jante

**Affiliations:** 1 Obstetrics and Gynaecology, North Eastern Indira Gandhi Regional Institute of Health and Medical Sciences (NEIGRIHMS), Shillong, IND

**Keywords:** amniotic fluid embolism, anaemia, dic, respiratory distress, first trimester mtp

## Abstract

Amniotic fluid embolism (AFE) may be a rare event in pregnancy, especially after a first-trimester medical termination of pregnancy (MTP). A 35-year-old G3P2L2 came to our outpatient department at six weeks of pregnancy for medical termination of pregnancy and bilateral tubal ligation. After around one hour of surgery, she developed respiratory distress with abdominal distension, hypotension, tachycardia and tachypnoea. On laparotomy, we found ascitic fluid, bowels with petechia, and oozing all over the wounds. Finally, within 24 hours of surgery, she expired. Strong clinical suspicion of AFE should prompt a multidisciplinary team including anaesthesia, respiratory therapy, critical care, and maternal-foetal medicine to be involved in the ongoing care of women with AFE.

## Introduction

Amniotic fluid embolism (AFE) is an uncommon, however frequently deadly situation precise to obstetrics. Current ideas concerning pathophysiology and control of this situation have been summarized elsewhere. The scientific expertise of this situation, its pathophysiology, and its control have all been traditionally hampered due to a loss of uniform diagnostic criteria [1]. The circumstance is characterized by the surprising onset of maternal compromise usually regarding the cardio-respiration and hematological systems, which could swiftly develop into cardiac arrest and profound coagulopathy to death [2]. Amniotic fluid embolism is common during later trimesters but here we are reporting a case of AFE during the first trimester of medical termination of pregnancy (MTP).

## Case presentation

**Figure 1:**
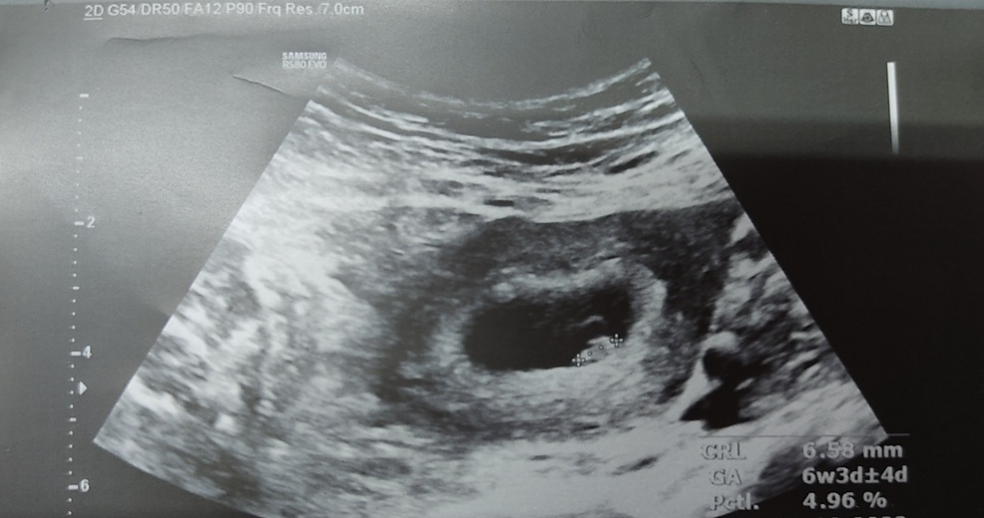
Ultrasonography image showing early intrauterine pregnancy

A 35-year-old G3P2L2 came to our outpatient department at six weeks of pregnancy (Figure 1) for MTP and bilateral tubal ligation. It was scheduled for an elective procedure in the operation theatre. A pre-operative investigation was performed ahead of surgery. Haemoglobin was 13gm per deciliter. Prothrombin time (PT), activated partial thromboplastin time (APTT) was within normal limit, complete blood count (CBC) was normal, and platelet count was >1.5lakh. Random blood sugar (RBS) was 110mg/dl. Suction evacuation and tubal ligation with a mini lap procedure were performed with fentanyl, midazolam and propofol in an operating room. Blood loss was around 100ml to 150ml. Foleys catheter was inserted. No hypoxia, tachycardia, or hypotension was noted in the operating room at the time of the procedure.

The patient was shifted to the post-operative room. After one hour she developed breathing difficulty with tachypnoea of respiratory rate of 30 breaths/minute. Oxygen saturation was 88% and the patient was started on oxygen. Gradually the patient deteriorated with hypotension of 90/60mmHg and tachycardia of 100 breaths/minute. She developed abdominal distension and was shifted to the ICU with haemoglobin of 5.4gm% and an altered coagulation profile with an international normalized ratio (INR) of 3. She was put on a ventilator and blood transfusion started. On ultrasound, the intraabdominal collection was seen and laparotomy was performed. A common diagnosis of uterine perforation was thought and a decision for laparotomy was taken. On laparotomy, there was ascitic fluid in the abdominal cavity and petechiae present all over the bowel, and oozing was present from all wound sites. Uterus was found normal with no perforation. No bleeding was observed from the tubal ligation site. The abdomen was closed. Thirteen units of packed blood cells were transfused along with fresh frozen plasma (FFP). Despite blood transfusion, the INR remained high. The patient deteriorated gradually and INR was increasing and not responding to inotropic agents. The patient expired the next morning. A post-mortem study could not be done as the patient's family refused. As the woman had respiratory distress, hypotension, disseminated intravascular coagulation (DIC) and cardiorespiratory collapse after the triggering event of a suction evacuation, the most probable diagnosis of amniotic fluid embolism was made.

## Discussion

Amniotic fluid embolism is an anaphylactoid syndrome of pregnancy (ASP) that usually develops during labour and leads to cardiorespiratory collapse and disseminated intravascular coagulation (DIC) [1]. There could also be an atypical presentation. Within the present case, however, cellular debris instead of the fluid intrinsically was the first agent which precipitated AFE and DIC. Amniotic fluid embolism may present in the absence of labour, but with medical or surgical abortion, spontaneous miscarriage, or obstetrical procedures including amniocentesis and amnioinfusion. The traditional myth goes that a woman in the final stages of labour becomes acutely dyspneic and hypotensive [1]. Amniotic fluid embolism has been reported to happen during an abortion, after abdominal trauma, amniocentesis, and amnioinfusion. To ensure that researchers utilise the same definition when reporting incidents, a consistent diagnostic criterion for amnionic fluid embolism has been proposed. For an AFE diagnosis, all of the following must be present [1]: (a) Cardiac arrest or both hypotension (systolic BP 90mm HG) and respiratory impairment (dyspnoea, oxygen saturation 90%); (b) documentation of overt DIC as a result of the circumstances described in item (a); (c) coagulopathy must be recognised before there is enough blood loss for dilutional or consumptive coagulopathy to occur; (d) clinical onset during labour or within half an hour after placenta delivery; (e) no fever (38.8 degrees Celsius) during labour.

The United Kingdom Obstetric Surveillance System (UKOSS) and the Australasian Maternity Outcomes Surveillance System (AMOSS) define AFE for case reporting as a condition characterised by the sudden onset of maternal compromise generally involving the cardio-respiratory and haematological systems, which can rapidly progress to cardiac arrest and profound coagulopathy leading to death [2,3].

Pulmonary embolism is often included within the medical diagnosis of an auto anticoagulated state and DIC of unknown aetiology but ascites could also be absent. Anaphylaxis reaction to any of the drugs might not be related to DIC. In our case, ascites are often explained as an inflammatory reaction within the peritoneum due to an anaphylactoid reaction. Sandhu et al. reported that post caesarean idiopathic ascites with no definitive cause, allergic or inflammatory peritoneal reaction could also be the foremost likely cause for this complication [4].

In the lack of any other possible explanation for the symptoms and signs observed, either a clinical diagnostic supported acute hypotension or asystole, acute hypoxia or coagulopathy, or a pathological/post-mortem diagnosis supported the presence of foetal squamous or hair within the lungs.

There has been no improvement in maternal survival since 1941 when AFE was first described [5,6]. Increased maternal age, inducement of labor, pregnancy, and placental abruption are all documented risk factors [7,8]. Low prevalence rates and selection bias continue to limit data. Few cases of AFE are documented within the trimester, both before and through uterine evacuation [9,10]. Clark et al. propose that foetal antigens reach the maternal circulation, generating a response similar to systemic inflammatory response syndrome (SIRS), with activation of the coagulation cascade leading to DIC and inflammatory-mediated myocardial function reduction [6]. Fekhkhar et al. in their study, describe the progression that usually occurs in two phases [9]. In the phase one clinical trial, hypoxia is caused by arteria pulmonalis vasospasm along with pulmonary hypertension and an increased right ventricular pressure. Myocardial and pulmonary capillary injury, left coronary failure, and acute respiratory distress syndrome, are all caused by hypoxia. Women who survive these occurrences may be eligible to participate in phase two clinical research. The initial presentation is generally a hemorrhagic phase with extensive bleeding, uterine atony, and DIC. However, deadly consumptive coagulopathy could also be the case [9].

Amniotic fluid embolism may be a rare and unpredictable event, with an incidence of roughly one in 40,000 deliveries and a reported death rate starting from 20% to 60% [9,10]. Early recognition of potential AFE and subsequent ICU management with supportive care including anaesthesia, respiratory therapy, critical care, and maternal-foetal medicine should be involved in the ongoing care of women with AFE.

## Conclusions

While it may be a rare event, strong clinical suspicion with the presence of hypotension, hypoxia, and coagulopathy after MTP in the first or second trimester, is the key to diagnosis. The foremost important aspect to the survival of patients with DIC and AFE is treatment with a timely multidisciplinary team approach. Publication and reporting of cases are important to enhance awareness, and hence reduce morbidity and mortality of those suspected of having AFE.
